# Age‐related positive emotional reactivity decline associated with the anterior insula based resting‐state functional connectivity

**DOI:** 10.1002/hbm.26621

**Published:** 2024-02-09

**Authors:** Lijing Niu, Xiaoqi Song, Qian Li, Lanxin Peng, Haowei Dai, Jiayuan Zhang, Keyin Chen, Tatia M. C. Lee, Ruibin Zhang

**Affiliations:** ^1^ Cognitive Control and Brain Healthy Laboratory, Department of Psychology, School of Public Health Southern Medical University Guangzhou China; ^2^ State Key Laboratory of Brain and Cognitive Sciences The University of Hong Kong Hong Kong SAR China; ^3^ Laboratory of Neuropsychology and Human Neuroscience The University of Hong Kong Hong Kong SAR China; ^4^ Center for Brain Science and Brain‐Inspired Intelligence Guangdong‐Hong Kong‐Macao Greater Bay Area Guangzhou China; ^5^ Department of Psychiatry, Zhujiang Hospital Southern Medical University Guangzhou PR China

**Keywords:** aging, anterior insula, emotional reactivity, functional connectivity

## Abstract

Recent studies have suggested that emotional reactivity changes with age, but the neural basis is still unclear. The insula may be critical for the emotional reactivity. The current study examined how ageing affects emotional reactivity using the emotional reactivity task data from a human sample (Cambridge Center for Age and Neuroscience, *N* = 243, age 18–88 years). The resting‐state magnetic resonance measurements from the same sample were used to investigate the potential mechanisms of the insula. In the initial analysis, we conducted partial correlation assessments to examine the associations between emotional reactivity and age, as well as between the gray matter volume (GMV) of the insula and age. Our results revealed that emotional reactivity, especially positive emotional reactivity, decreased with age and that the GMV of the insula was negatively correlated with age. Subsequently, the bilateral insula was divided into six subregions to calculate the whole brain resting‐state functional connectivity (rsFC). The mediating effect of the rsFC on age and emotional reactivity was then calculated. The results showed that the rsFC of the left anterior insula (AI) with the right hippocampus, and the rsFCs of the right AI with the striatum and the thalamus were mediated the relationship between positive emotional reactivity and age. Our findings suggest that attenuating emotional reactivity with age may be a strategic adaptation fostering emotional stability and diminishing emotional vulnerability. Meanwhile, the findings implicate a key role for the AI in the changes in positive emotional reactivity with age.

## Graphical abstract

Emotional reactivity decreases with age, especially positive emotional reactivity, the resting‐state functional connectivity of the anterior insula with the striatum mediated the positive emotional reactivity and age.

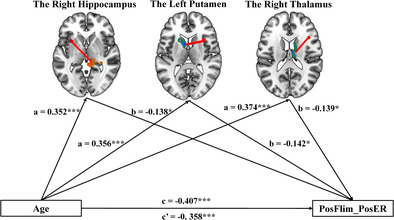

## 1 INTRODUCTION

The steady advancement of public health and medical care has yielded substantial improvements in global longevity (Kemoun et al., 2022). Nevertheless, the aging process is accompanied by pervasive challenges, including compromised health status (Idler & Benyamini, 1997; Salthouse, 2005), diminished physical function (Fried et al., 2001), and cognitive function decline (Mendes, 2010). Interestingly, the emotional well‐being of older adults presents a paradoxical picture. In contrast to the anticipated decline in other cognitive domains (Mather, 2012), emotional health often thrives in later life, marked by enhanced emotional stability, reduced emotional variability, and decreased reactivity to daily stressors (Brose et al., 2013; Burr et al., 2021). Emotional reactivity, is an important component of emotional health (Brose et al., 2013), and research into how emotional reactivity changes with age holds great promise for enhancing our understanding of mental health across the human lifespan.

Recent studies have employed socioemotional selectivity theory (SST) (Carstensen et al., 1999) in trying to resolve the paradox. SST suggests that as future time horizons typically shorten with age, emotional goals are prioritized over goals that focus on exploration. As the time remaining becomes more limited, positive messages are remembered better than negative ones. Some studies support this view, and have shown that as individuals age, they tend to focus less on negative emotions (Carstensen & Mikels, 2005; Mather & Knight, 2005; Scheibe & Carstensen, 2010; Smith et al., 2005) while being more motivated to sustain positive affectivity (Riediger et al., 2009). However, other studies have found no age‐related changes in emotional reactivity (Admon & Pizzagalli, 2015) or a decline in reactivity with age (Backs et al., 2005). One possible reason for these inconsistent results could be the instability of emotional reactivity elicited by pictorial stimuli. Research has shown that natural stimuli, such as well‐directed films, elicit and manipulate our feelings and emotions more effectively (Guo et al., 2015; Jääskeläinen et al., 2008). Therefore, the use of films as task stimuli may lead to more stable emotional reactivity in individuals, thereby avoiding to some extent inconsistencies in results.

Interoceptive sensitivity (IS) takes center stage in this exploration, representing the capacity to accurately apprehend visceral and afferent information, thereby reflecting an individual's physiological awareness (Garfinkel & Critchley, 2013). Grounded in peripheral theories of emotion, emotional reactivity is believed to hinge on physiological arousal (Damasio, 1999; James, 1884). IS occupies a pivotal position within this paradigm, as experiments employing pharmacological blockade to mitigate peripheral changes have demonstrated a commensurate reduction in emotional reactivity, particularly for negative, high‐arousal emotions under stress (MacCormack et al., 2020). This connection is reinforced by findings that individuals with greater interoceptive ability, adept at discerning ongoing visceral changes, tend to experience more intense and highly aroused emotions (Schulz & Vögele, 2015). A body of empirical evidence suggests that IS diminishes with age (Kenney & Chiu, 2001; Lautenbacher et al., 2017; Rayner et al., 2000), with older adults displaying reduced sensitivity to physiological changes precipitated by emotional stimuli (Ulus & Aisenberg‐Shafran, 2022). Based on the above research, it is hypothesized that the intensity of emotional reactivity decreases with age.

Among the regions exhibiting activation related to interoceptive awareness, the insular cortex has been identified as a crucial neural substrate for integrating information from interoception and subjective emotional reactivity (Critchley et al., 2004). According to the neural cognitive somatic marker model, the insula mediates the reception of interoceptive and body state signals, which are translated into subjective feelings, emotional reactivity, and the self‐awareness of urges (Craig, 2002; Craig, 2010; Damasio, 1999; Stone, 2000). In essence, the insula not only processes interoceptive activity but also integrates and modulates cardiovascular, respiratory, and emotional signals to generate integrated emotional reactivity. Therefore, as a hub of the interoceptive awareness system (Zhao et al., 2023), the insula may play a crucial role in emotional reactivity.

The insula is a complex region consisting of structurally and functionally distinct subregions (Deen et al., 2011; Ichesco et al., 2014), which can be categorized into the anterior insula (AI), middle insula (MI), and posterior insula (PI) (Taylor et al., 2009). These subregions contribute differentially to emotional processing with the AI chiefly orchestrating the integration of cognitive, emotional, and sensory processes to engender subjective perceptions and understanding of emotional states (Gu et al., 2013; Wager & Barrett, 2004). Therefore, the AI may hold greater relevance for emotional reactivity. In contrast, the MI/PI primarily oversees interoception and interoceptive attention, focusing on internal states (Farb et al., 2013; Haruki & Ogawa, 2021; Simmons et al., 2013). The PI typically encodes somatosensory inputs, which are then harmonized with limbic/emotional information in the MI, and ultimately integrated with cognitive processes in the AI (Centanni et al., 2021). One study has unearthed differences between younger and older adults in functional brain activation during affect induction and perception tasks. Younger individuals displayed more pronounced activation in the PI, whereas older individuals exhibited heightened activation in the AI (MacCormack et al., 2020). This divergence could be attributed to younger individuals' heightened engagement with visceral signals during emotional reactivity compared to older adults, who tend to be more occupied with processes such as mentalizing, autobiographical memory, and self‐regulation. We therefore hypothesize that AI becomes increasingly important in emotional reactivity with age.

Despite the extensive exploration of age‐related changes within the insula, the focus has primarily been on structural alterations, as reflected in the age‐related decline in insular cortical thickness and gray matter volume (GMV; Churchwell & Yurgelun‐Todd, 2013; Good et al., 2001; Lamballais et al., 2020; Myoraku et al., 2022; Shepherd et al., 2012). Notably, the relationship between structural changes in the insula and emotional reactivity remains largely uncharted. Furthermore, age‐related changes in insula‐based functional connectivity have been documented in older adults (Ai et al., 2022). Resting‐state functional connectivity (rsFC) emerges as an invaluable tool for comprehending age‐related brain transformations, enabling the examination of intrinsic functional networks devoid of the confounding influence of task‐related activities (Geerligs et al., 2015). The rsFC presents a potential avenue for unraveling the intricacies of the aging process and its association with declining emotional reactivity.

The current study aims to explore the changes in emotional reactivity with age and to investigate whether the rsFC of insula subregions is involved. The study utilized a laboratory‐based emotional reactivity and regulation task with films as the stimuli (Schweizer et al., 2013; Schweizer et al., 2016), the aim being to examine age‐related changes in emotional reactivity triggered under passive viewing and different emotion regulation conditions. We used a data sample of adults aged 18–88 years (*N* = 243) from the Cambridge Center for Age and Neuroscience (Cam–CAN) cohort (Shafto et al., 2014) (https://www.cam-can.or). First, we employed the data from an emotional reactivity and regulation task to test the hypothesis that emotional reactivity declines with age. Next, we analyzed whether age‐related changes in the structure of the insula were also associated with emotional reactivity using voxel‐based morphometry (VBM) analysis. Then, based on resting‐state magnetic resonance (MR) measurements, we identified six regions of interest in the bilateral insula (Zhang et al., 2022). Subsequently, mediation models were developed to explore whether the rsFC of insula subregions, which were found to be significantly associated with age, mediated the relationship between age and emotional reactivity.

## 2 MATERIALS AND METHODS

### 2.1 Participants

**FIGURE 1:**
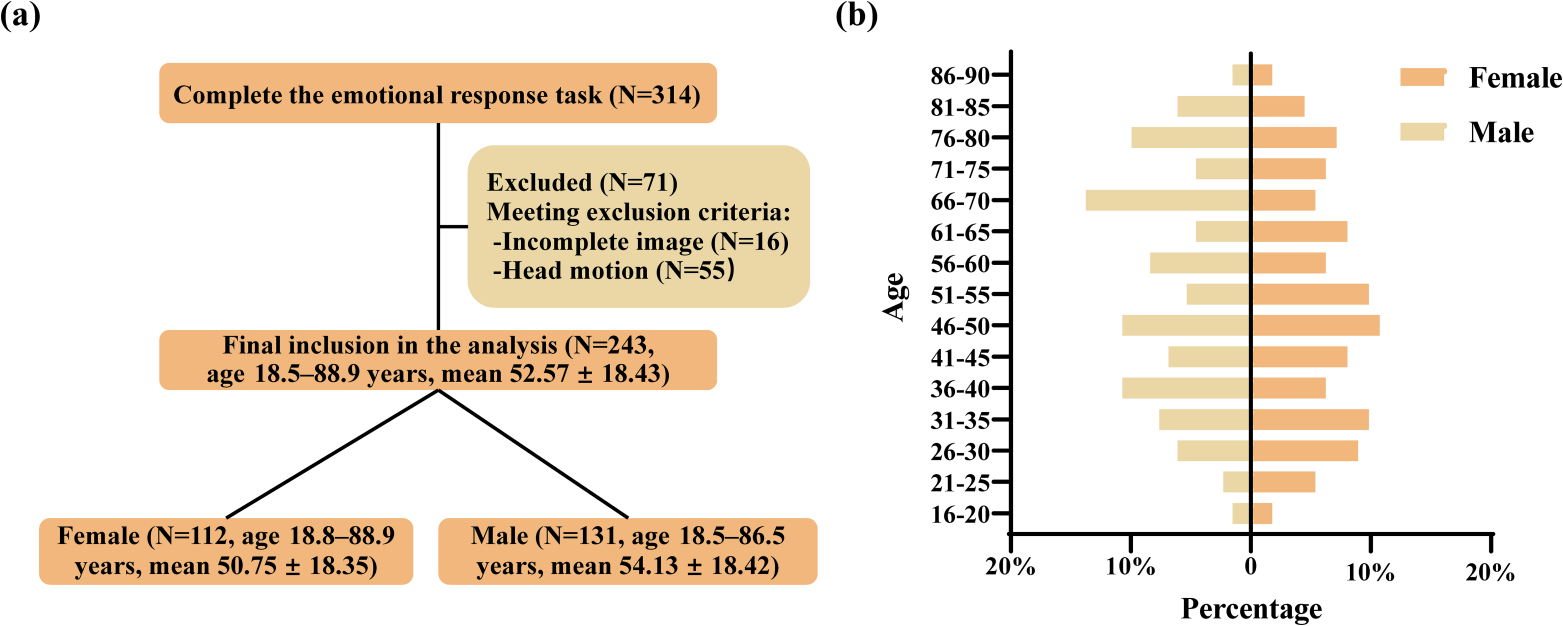
Individuals flow diagram and sample age distribution. (a) The inclusion criteria of the participants and the number of participants included in the final analysis. (b) The 243 participants retained from the Cambridge Center for Age and Neuroscience (Cam–CAN) database sufficiently spanned ages 18–88, and there were no significant differences in age between the genders of the participants (*t* = −1.427, *p* = .155).

Data used in the present study came from the Cambridge Centre for Ageing and Neuroscience (Cam–CAN) (https://www.cam-can.or), which is a large‐scale, population‐based adult lifespan dataset aiming at uncovering the neural underpinnings of ageing (Taylor et al., 2017). All participants in this Cam–CAN project demonstrated cognitive health, were free from severe mental illnesses, and qualified for MRI scans. Detailed exclusion criteria are provided in the supplementary materials. Out of the Cam–CAN cohort (Shafto et al., 2014), 330 individuals were invited to perform an emotional reactivity and regulation task, with 16 individuals opting not to participate. Additionally, we excluded 16 participants who did not complete the fMRI scan, and 55 participants because of problems analyzing their MR images (head motion >2.5 mm of translation or 2.5° of rotation, mean framewise displacement [FD] >0.50) (Figure 1a). Overall, a total of 243 participants were included in the subsequent analysis for the study (age 18.5–88.9 years, mean 52.57 ± 18.43). Among them, there were 112 females (age 18.8–88.9 years, mean 50.75 ± 18.35) and 131 males (age 18.5–86.5 years, mean 54.13 ± 18.42), with no significant gender differences in age (*t* = −1.427, *p* = .155) (Figure 1b). Ethical approval for the Cam–CAN study was obtained from the Cambridge Shire 2 (now East of England–Cambridge Central) Research Ethics Committee, and written informed consent was obtained from each participant.

### 2.2 Emotional reactivity and regulation task

**FIGURE 2:**
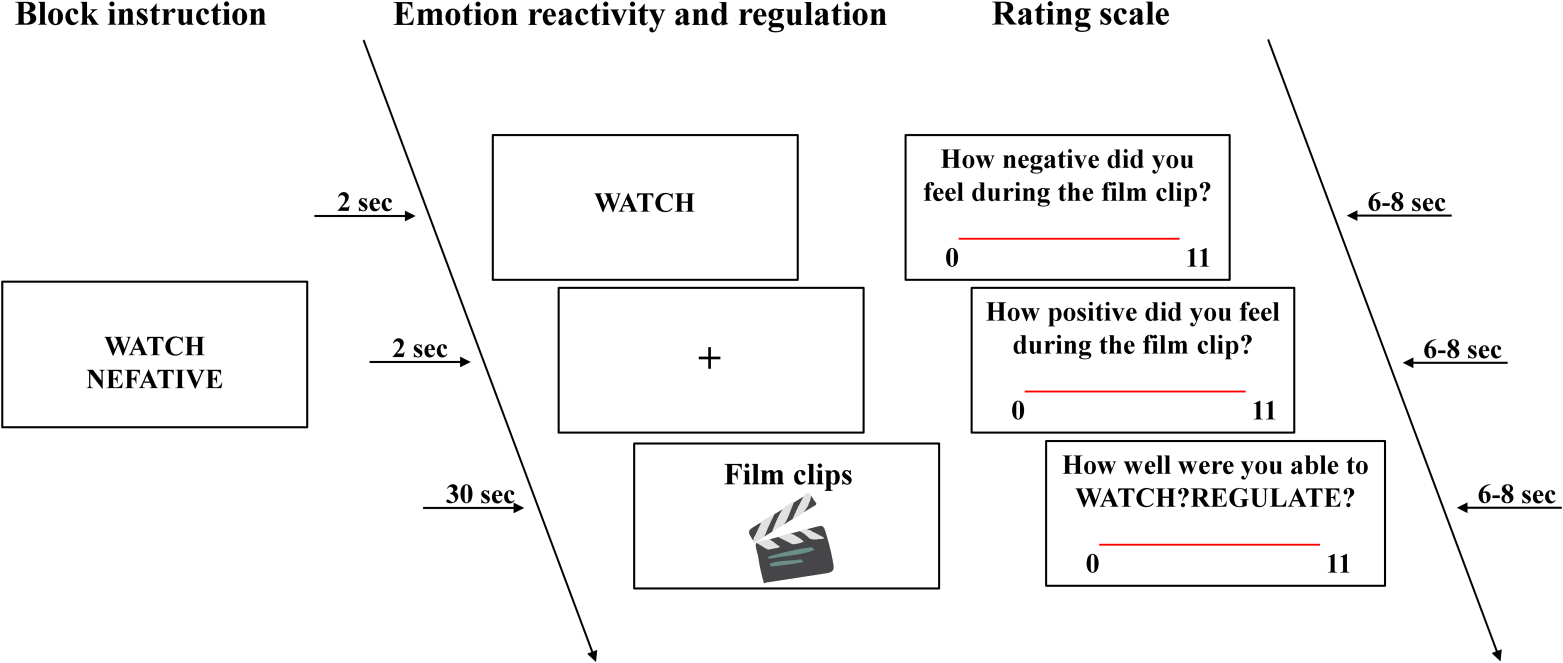
Emotion reactivity and regulation task. In the WATCH condition (example in the above figure), participants were asked to simply watch the films and allow their emotions to arise naturally. In the REGULATE condition, participants were asked to reappraise their emotions about a negative film by changing the way they thought about the content of the film. After each film clip, participants rated the positive and negative effects they felt during the film on two valence scales and their compliance with the task instructions.

Emotional reactivity and regulation were assessed under a film‐based paradigm (Figure 2) (Schweizer et al., 2013; Schweizer et al., 2016). Participants viewed 30‐s film clips across four different conditions: watch neutral, watch positive, watch negative, and reappraise negative. In the watch conditions, they were instructed to simply watch the films and allow their emotions to arise naturally. In the reappraise condition they were asked to reduce their negative emotions by changing the way they think about the content of the films. After each film clip, they were instructed to rate their emotions during the film. Ratings were provided on two rating scales measuring positive and negative emotions, ranging from 1 (not at all) to 11 (extremely).

Participant ratings served as a behavioral index of emotional reactivity and emotional regulation effectiveness. Emotional reactivity was computed using the following equations, with higher scores indicating higher emotional reactivity:

(1) $$\text{PosFilm}\_\text{PosER}=\left({{\text{Positive}}^{\text{Watch}}}_{\text{positive}}-{{\text{Neutral}}^{\text{Watch}}}_{\text{positive}}\right)$$

(2) $$\text{PosFilm}\_\text{NegER}=\left({{\text{Positive}}^{\text{Watch}}}_{\text{negative}}-{{\text{Neutral}}^{\text{Watch}}}_{\text{negative}}\right)$$

(3) $$\text{NegFilm}\_\text{PosER}=\left({{\text{Negative}}^{\text{Watch}}}_{\text{positive}}-{{\text{Neutral}}^{\text{Watch}}}_{\text{positive}}\right)$$

(4) $$\text{NegFilm}\_\text{NegER}=\left({{\text{Negative}}^{\text{Watch}}}_{\text{negative}}-{{\text{Neutral}}^{\text{Watch}}}_{\text{negative}}\right)$$

For the four task conditions, PosFilm_PosER represents the positive emotional reactivity to watching positive films, PosFilm_NegER represents the negative emotional reactivity to watching positive films, NegFilm_PosER represents the positive emotional reactivity to watching negative films, and NegFilm_NegER represents the negative emotional reactivity to watching negative films. Positive emotion regulation of negative films (NegFilm_PosRegulation) and negative emotion regulation of negative films (NegFilm_NegRegulation) were computed using the following equations, with higher scores indicating better emotion regulation ability:

(5) $$\text{NegFilm}\_\text{PosRegulation}=\left({{\text{Negative}}^{\text{regulate}}}_{\text{positive}}-{{\text{Negative}}^{\text{Watch}}}_{\text{positive}}\right)$$

(6) $$\text{NegFilm}\_\text{NegRegulation}=\left({{\text{Negative}}^{\text{regulate}}}_{\text{negative}}-{{\text{Negative}}^{\text{Watch}}}_{\text{negative}}\right)$$

To obtain the relationship between emotional reactivity and age, as well as the relationship between emotion regulation effectiveness and age, we conducted a partial correlation analysis, with gender as a covariate. The choice to use gender as the sole covariate was because we only obtained gender information. Additionally, we referred to other studies using the Cam–CAN dataset, which utilized gender, education level, and intelligence as covariates but found no significant impact (Schweizer et al., 2019; Stretton et al., 2022).

### 2.3 Image data acquisition and processing

Details of the fMRI data acquisition can be found in the supplementary materials. Briefly, resting‐state scans were collected while participants rested with their eyes closed. Preprocessing of resting‐state fMRI data was performed using Graph Theoretical Network Analysis (GRETNA; http://www.nitrc.org/projects/gretna/) (Wang et al., 2015), which included the following: (1) removal of the first five time points for steady magnetization and participant adaptation; (2) slice timing correction; (3) head motion correction; (4) performing spatial normalization to the Montreal Neurological Institute (MNI) space by applying new segments to the structural images (resampling voxel size = 3 mm × 3 mm × 3 mm); (5) spatial smoothing using an isotropic Gaussian kernel of 6 mm full width at half maximum (FWHM); (6) removal of the linear drift within the time series; (7) regressing out the effects of head movement (Friston 24 parameter model), cerebrospinal fluid (CSF) signal noise, and white matter (WM) signal noise from the fMRI data; and (8) band‐pass filtering (0.01–0.1 Hz). With regard to global signal regression, first, we calculated the Global Negative Index (GNI) as an indicator of whether or not the current study required global signal regression (Chen et al., 2012). Results of the GNI calculation for each subject indicated that more than half the participants had a GNI greater than 3, which according to Chen et al.'s recommendations (Chen et al., 2012) indicated that global signal regression was not required in the current study. Second, we also calculated the partial correlation between the whole brain signal and age (*r* = −.105, *p* = .104), with head movement profile and sex as covariates. As a consequence of these calculations, we did not perform global signal regression on this dataset. The subjects' head motion during fMRI sessions had a positive, linear relationship with age, so the head motion was controlled for in subsequent analyses.

### 2.4 Voxel‐based morphometry

For VBM analysis of imaging data, we used the Computational Anatomy Toolbox (CAT12; C. Gaser, Structural Brain Mapping Group, Jena University Hospital, Jena, Germany, http://www.neuro.uni-jena.de/cat/) is implemented in SPM12 (Statistical Parametric Mapping, Institute of Neurology, London, UK). All T1‐weighted images were corrected for bias‐field inhomogeneities, and then segmented into gray matter (GM), WM, and CSF. After preprocessing and in addition to visual checks for artifacts, all scans passed an automated quality check protocol. Scans were smoothed with a Gaussian kernel of 8 mm (FWHM). We used the AAL90 atlas (Tzourio‐Mazoyer et al., 2002) to extract the GMV of the left and right insula as regions of interest. We then performed partial correlations between GMV with age and emotional reactivity, respectively, and with total intracranial volume (TIV) and gender as covariates to remove the related variance.

### 2.5 Functional connectivity and statistical analyses

**FIGURE 3:**
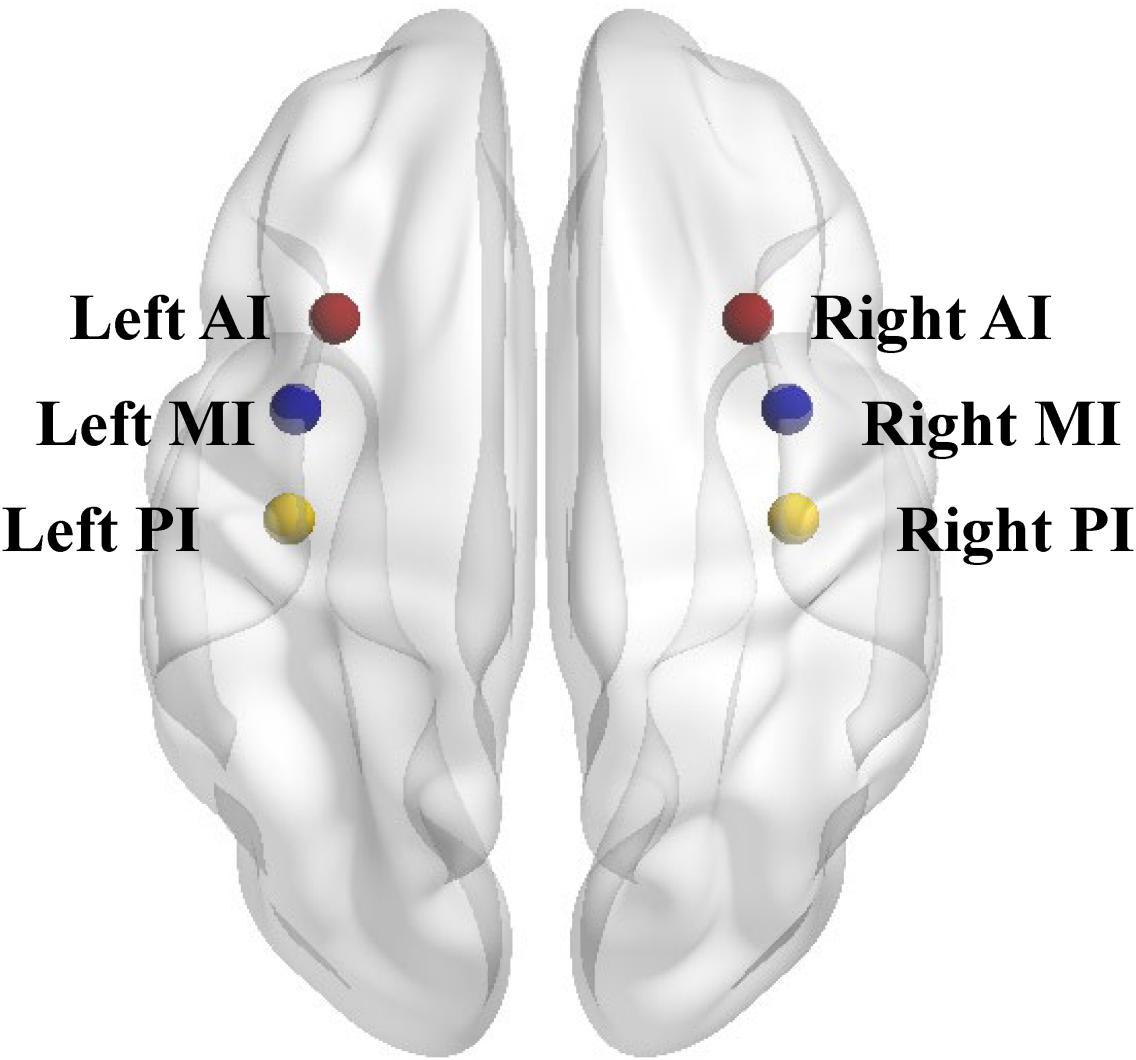
Insula subregions in Montreal Neurological Institute (MNI) standard space. The picture shows insula subregions seeds in the MNI standard space, including the left AI (MNI = −32, 16, 6; red); the left MI (MNI = −38, 2, 8; blue); the left PI (MNI = −39, −15, 1; yellow); the right AI (MNI = 32, 16, 6; red); the right MI (MNI = 38, 2, 8; blue); and the right PI (MNI = 39, −15, 8; yellow). AI = anterior insula; MI = middle insula; PI = posterior insula.

To examine the rsFC of insula subregions implicated in emotional reactivity and age, six spherical seeds with a 6 mm radius were defined and centered on six MNI coordinates: the left AI (MNI = −32, 16, 6); the right AI (MNI = 32, 16, 6); the left MI (MNI = −38, 2, 8); the right MI (MNI = 38, 2, 8); the left PI (MNI = −39, −15, 1); and the right PI (MNI = 39, −15, 8) (Figure 3). The coordinates of the six seeds were derived from a previous study by Taylor (Taylor et al., 2009), which have been widely applied (Chen et al., 2020; Zhang et al., 2022; Zhao et al., 2017).

Subsequently, seed‐based rsFC analyses were performed with the following steps. For each subject, we calculated the correlation coefficient between the averaged time course of each subregion and the time courses of all other brain voxels. To assess and compare the rsFC, we converted these image maps, which were not normally distributed, to *z*‐score maps by Fisher's *r*‐to‐*z* transform (Xiao et al., 2018). Next, using mean FD and gender as covariates, partial correlation analysis was conducted between the *Z* map and age to obtain rsFC significantly correlated with age. The rsFC between voxel clusters and the insula subregions was considered to have a statistically significant correlation with age (Gaussian random‐field [GRF] correction with voxel *p* < .001, cluster *p* < .05). The peak voxel within each cluster was identified as the representative of that cluster.

### 2.6 Mediation analysis

To assess the relationship between age, emotional reactivity, and the rsFCs of insula subregions, the following steps were undertaken. First, we used the cluster of rsFCs significantly correlated with age as a mask to extract the *z*‐values of each voxel for averaging, and then obtained the mean rsFC value of each cluster for each participant. Second, with age as the independent variable, emotional reactivity as the dependent variable, and the mean rsFC value between the subregions and the significant cluster as the mediating variable, we performed mediation analyses that used stepwise regression and bootstrap methods to further examine the association between age, emotional reactivity, and the rsFCs of insula subregions. For the optimum test of the mediation effect, the bootstrapping procedure to measure the indirect effect was carried out and 95% confidence intervals were estimated. The number of bootstrap samples was 5000. Gender and mean FD were included as covariates in the model. If the confidence interval includes zero, it means that there is no significant indirect mediating effect at the significance level of 5%. All statistical analyses were performed using SPSS 25.0 (SPSS Inc., Chicago, Illinois, USA). The mediation model was analyzed using Model 4 in the PROCESS Marco (Montoya & Hayes, 2017).

## 3 RESULTS

### 3.1 The intensity of emotional reactivity decreased with age

**FIGURE 4:**
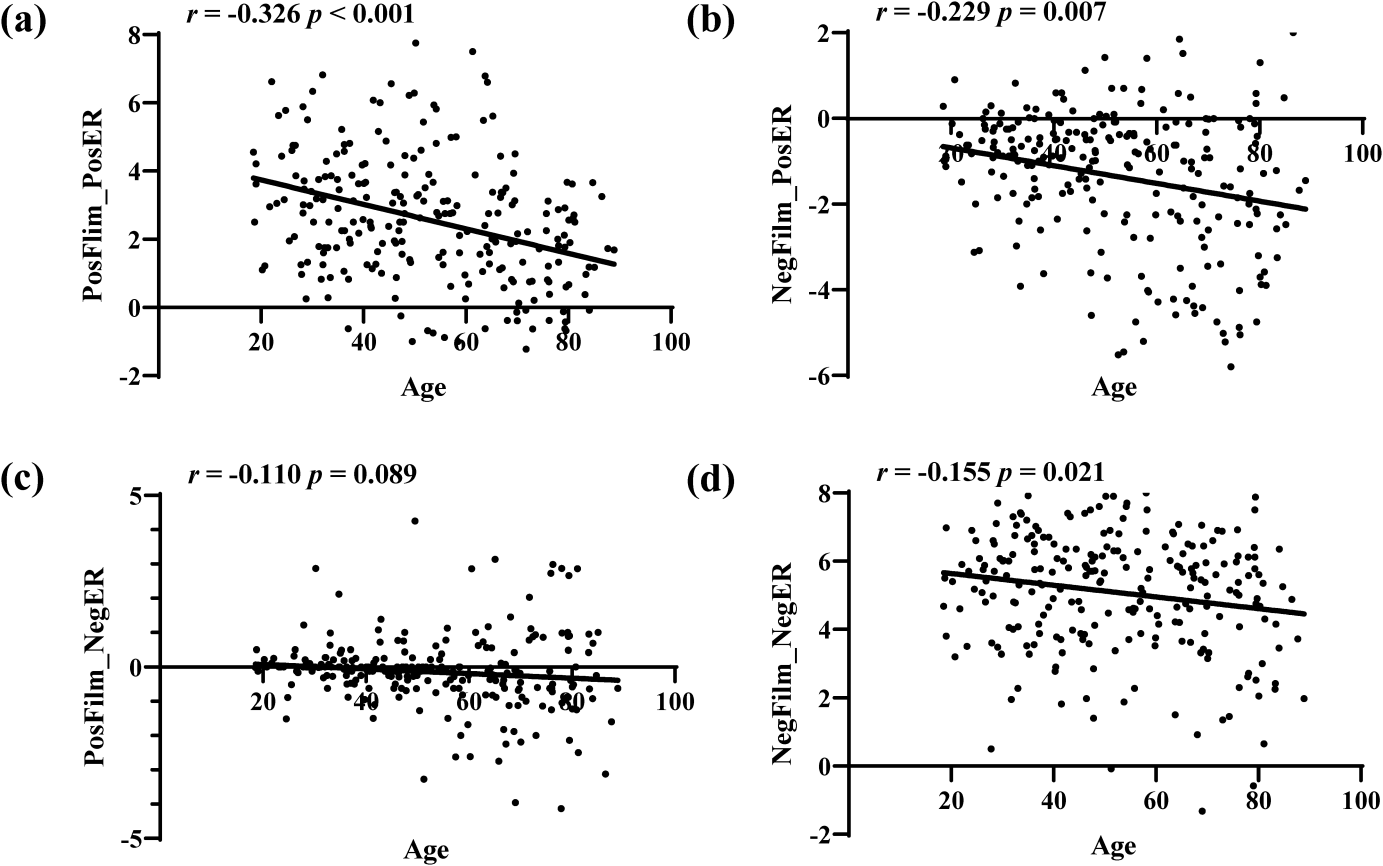
Emotional reactivity decreased with age. Scatterplots depicting the correlations between age and emotional reactivity after control gender and mean FD. (a, b) The positive emotional reactivity decreased with age; (c) the negative emotional reactivity to positive stimuli shows a certain downward trend but the correlation is not significant; and (d) the negative emotional reactivity to negative stimuli decreased with age.

To determine if the individual intensity of emotional reactivity decreased with age, the correlation between the intensity of emotional reactivity and age was assessed using partial correlation analysis, with gender as a covariate (Figure 4). The results showed that age was significantly correlated with the negative reactivity to negative films (*r* = −.155, *p* = .016, FDR‐corrected *p* = .021), the positive reactivity to negative films (*r* = −.229, *p* = .003, FDR‐corrected *p* = .007), and the positive reactivity to positive films (*r* = −.326, *p* < .001, FDR‐corrected *p* < .001). There was no significant correlation between the negative reactivity to positive films and age (*r* = −.110, *p* = .089, FDR‐corrected *p* = .089), but there was also a downward trend (Figure 4b). However, the effect of positive emotion regulation (*r* = .018, *p* = .786) and the effect of negative emotion regulation (*r* = −.19, *p* = .768) were not significantly related to age.

### 3.2 The GMV of the insula was negatively correlated with age

**FIGURE 5:**
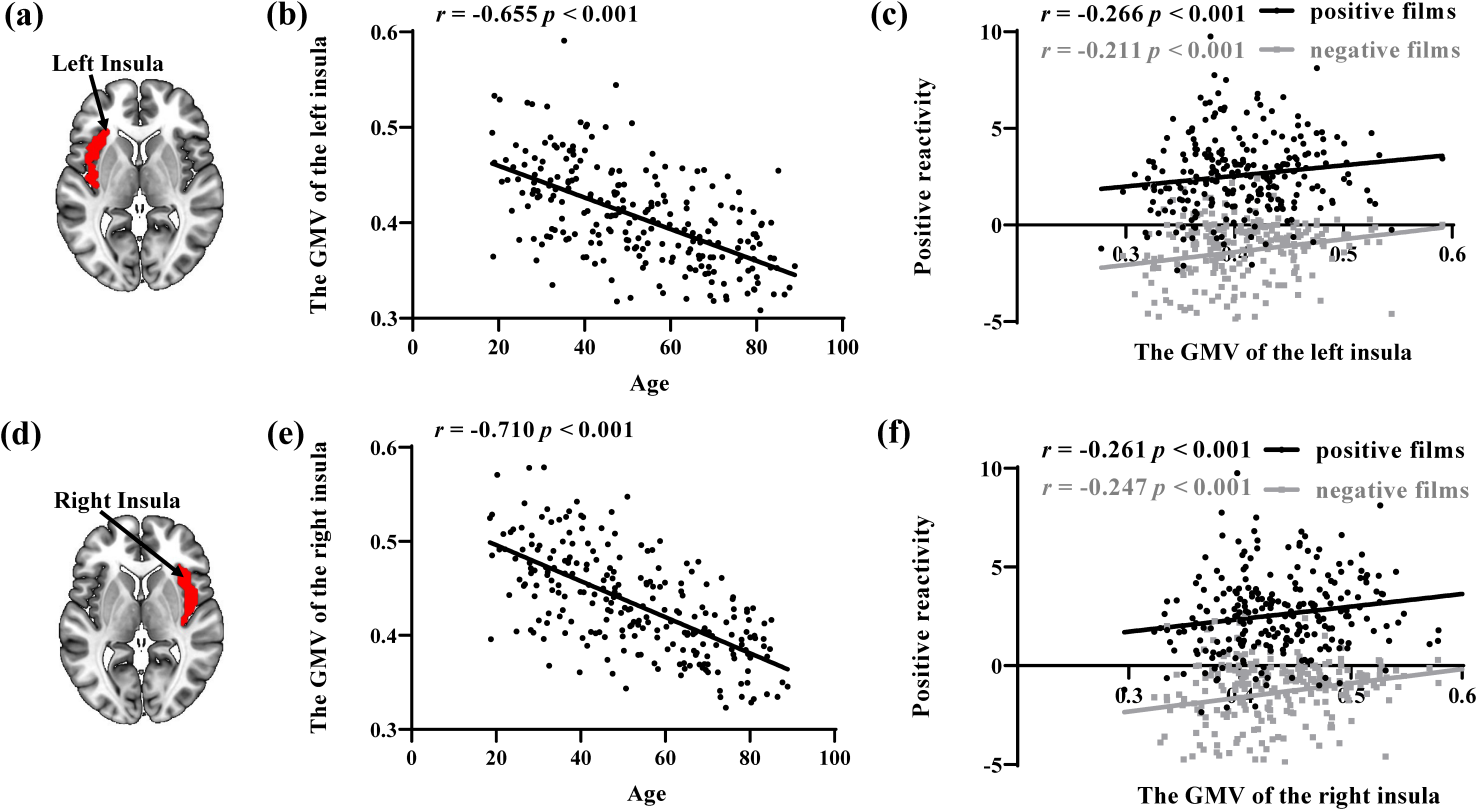
Results of the voxel‐based morphometry (VBM) analysis. (a, d) The left/right insula in the AAL atlas; (b, e) the gray matter volume (GMV) of the bilateral insula decreased significantly with age; and (c, f) the GMV of the bilateral insula increased with positive emotional reactivity. Partial correlation analyses all included gender, head movement, and total intracranial volume (TIV) as covariates. L, left hemisphere; R, right hemisphere.

The VBM analysis showed that the GMV of both the left and right insula were negatively correlated with age (*r*_*left insula*_ = −.655, *p* < .001, FDR‐corrected *p* < .001; *r*_*right insula*_ = −.710, *p* < .001, FDR‐corrected *p* < .001) (Figure 5b,e). The GMV of both the left and right insula were significantly positively correlated with the positive reactivity to watching positive films (*r*_*left insula*_ = .226, *p* < .001, FDR‐corrected *p* < .001; *r*_*right insula*_ = .261, *p* < .001, FDR‐corrected *p* < .001) and the positive reactivity to watching negative films (*r*_*left insula*_ = .211, *p* < .001, FDR‐corrected *p* < .001; *r*_*right insula*_ = .247, *p* < .001, FDR‐corrected *p* < .001) (Figure 5c,f). However, there was no significant correlation with the negative reactivity. Further mediation analysis did not find that the GMV of the left and right insula mediated the relationship between age and positive emotional reactivity.

### 3.3 Age‐related rsFCs of the insula

#### 3.3.1 The AI

**FIGURE 6:**
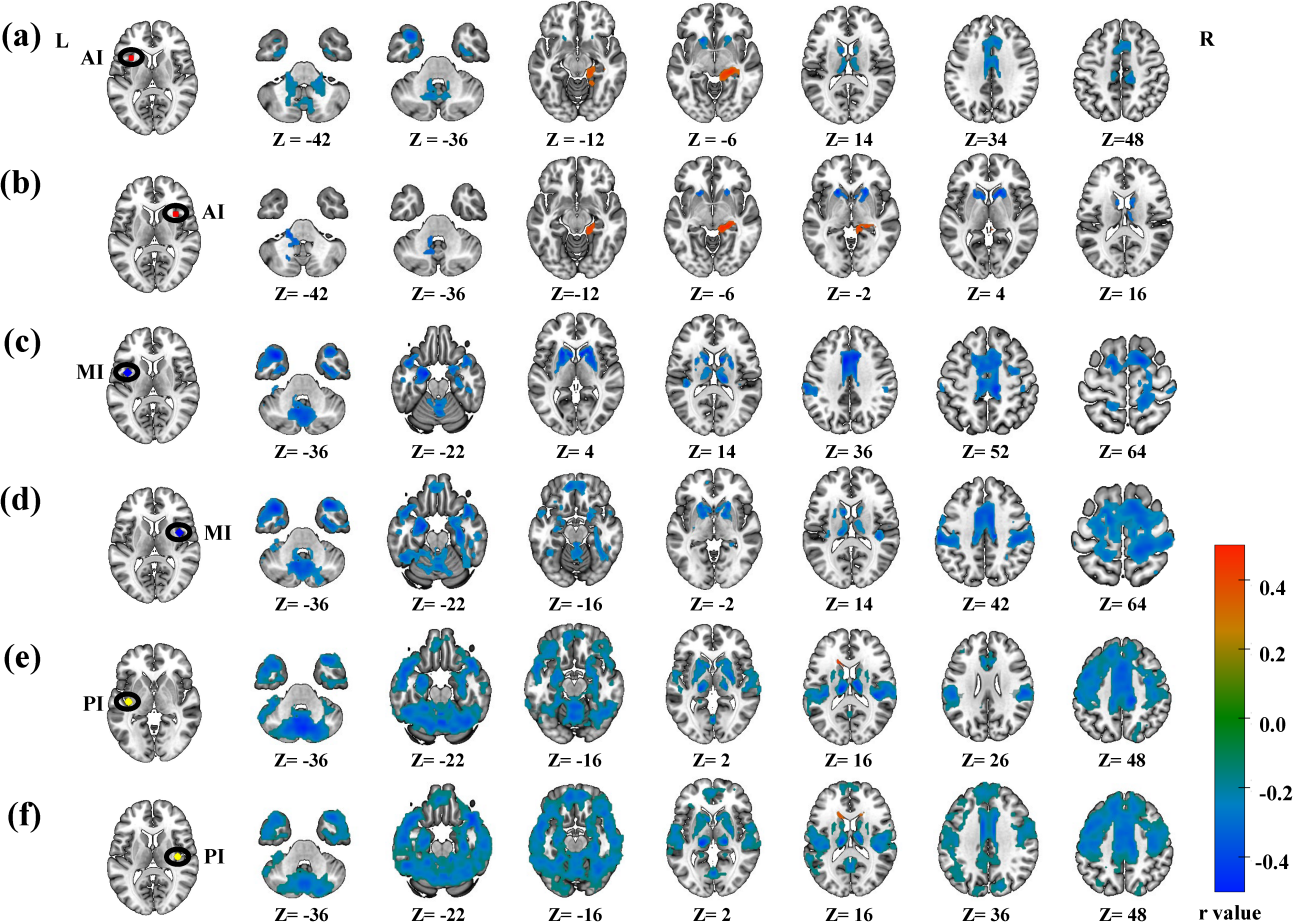
Age‐related whole brain functional connectivity in the insula subregions. Whole brain functional connectivity was significantly associated with age in six insula subregions (corrected for GRF, voxel *p* < .001, cluster *p* < .05, family‐wise error). Clusters with positive and negative connectivity are shown in warm and cool colors, respectively. L, left hemisphere; R, right hemisphere.

After applying GRF correction, seven clusters of the left AI and four clusters of the right AI remained significantly correlated with age. To be specific, the rsFC between the left AI and the right hippocampus increased with age, whereas the rsFC between the vermis, bilateral thalamus, bilateral fusiform nucleus, and right cingulate and the left AI decreased (Figure 6a, Table 1). Interestingly, we also observed the reduced rsFC with age between the right AI and the inferior cerebellum, the right thalamus, and the left putamen, as well as an increased rsFC between the right AI and the right hippocampus (Figure 6b, Table 1).

**TABLE 1:** Regions showing the age effect of the functional connectivity for the insula subregions.

Number of voxels	Peak intensity	MNI coordinate			Side	Identified brain regions (AAL atlas)
		*x*	*y*	*z*		
The left AI (positive correlation with age)						
190	0.366	24	−30	−3	R	Hippocampus
The left AI (negative correlation with age)						
438	−0.339	6	−51	−27	‐	Vermis
118	−0.304	33	−9	−36	R	Fusiform
350	−0.408	−36	12	−33	L	The middle temporal gyrus
279	−0.418	−9	−21	18	L	Thalamus
231	−0.478	6	−9	18	R	Thalamus
725	−0.382	9	−12	−33	R	The medial and lateral cingulate gyri
The right AI (positive correlation with age)						
109	0.301	24	−30	−6	R	Hippocampus
The right AI (negative correlation with age)						
108	−0.313	−15	−36	−42	L	The inferior cerebellum
118	−0.304	−21	21	−3	L	Putamen
164	−0.371	6	−9	18	R	Thalamus
The left MI (positive correlation with age)						
‐	‐	‐	‐	‐	‐	‐
The left MI (negative correlation with age)						
915	−0.425	9	9	9	R	Caudate
1459	−0.406	−18	−6	−24	R	ParaHippocampal
433	−0.397	−6	9	0	L	Caudate
173	−0.418	−9	−21	18	R	Heschl
252	−0.333	−63	−27	42	L	The supramarginal gyrus
1968	−0.442	−12	6	42	L	The medial and lateral cingulate gyri
The right MI (positive correlation with age)						
‐	‐	‐	‐	‐	‐	‐
The right MI (negative correlation with age)						
3974	−0.475	42	15	−27	R	The superior temporal gyrus
224	−0.323	−9	51	−18	L	Rectus
393	−0.384	−9	6	12	L	Caudate
4656	−0.416	18	−24	45	R	The medial and lateral cingulate gyri
The left PI (positive correlation with age)						
‐	‐	‐	‐	‐	‐	‐
The left PI MI (negative correlation with age)						
18,905	−0.475	−18	−24	12	L	Thalamus
The right PI (positive correlation with age)						
‐	‐	‐	‐	‐	‐	‐
The right PI (negative correlation with age)						
24,826	−0.475	−18	−24	12	L	Thalamus

*Note*: GRF correction uses voxel *p* < .001, cluster *p* < .05, family‐wise error. R, right hemisphere; L, left hemisphere.

#### 3.3.2 The MI

After GRF correction, six clusters with the left MI and four with the right MI were significantly negatively correlated with age. The results showed decreased rsFCs with age between the left MI and the left supramarginal, bilateral caudate, left parahippocampal, right heschl, and left cingulum (Figure 6c, Table 1). The rsFCs between the right MI and the bilateral caudate, left parahippocampal, right heschl, left supramarginal, and left cingulum also decreased with age (Figure 6d, Table 1). There were no clusters of significantly increased rsFC with the bilateral MI (Figure 6c,d, Table 1).

#### 3.3.3 The PI

Unlike AI and MI, all the voxels with significant age effects in the rsFCs of the left and right PI were identified within one cluster, and all the rsFCs decreased with age (Figure 6e,f, Table 1).

### 3.4 The rsFC of the AI mediated the relationship between age and emotional reactivity

The results showed that the rsFC of the left AI with the right hippocampus cluster, as well as the rsFCs of the right AI with the left putamen cluster and the right thalamus cluster, significantly mediated the relationship between age and positive emotional reactivity to watching positive films. Specifically, controlling for gender and mean FD, age had a significant relationship with positive emotional reactivity to watching positive films (*β* = −.407, *p* < .001). Second, controlling for gender and mean FD, age had a significant positive correlation with the rsFC of the left AI with the right hippocampus cluster (*β*_*hippocampus*_ = .352, *p* < .001, Table S2), and significant negative associations with the rsFCs of the right AI with the left putamen cluster and the right thalamus cluster (*β*_*Putamen*_ = −.356, *p* < .001, Table S3; *β*_*Thalamus*_ = −.374, *p* < .001, Table S4). Finally, controlling for gender and mean FD, both age and rsFC were included in the mediation model and showed a significant relationship with positive emotional reactivity to watching positive films (Tables S2, S3, and S4 in the supplementary materials).

**FIGURE 7:**
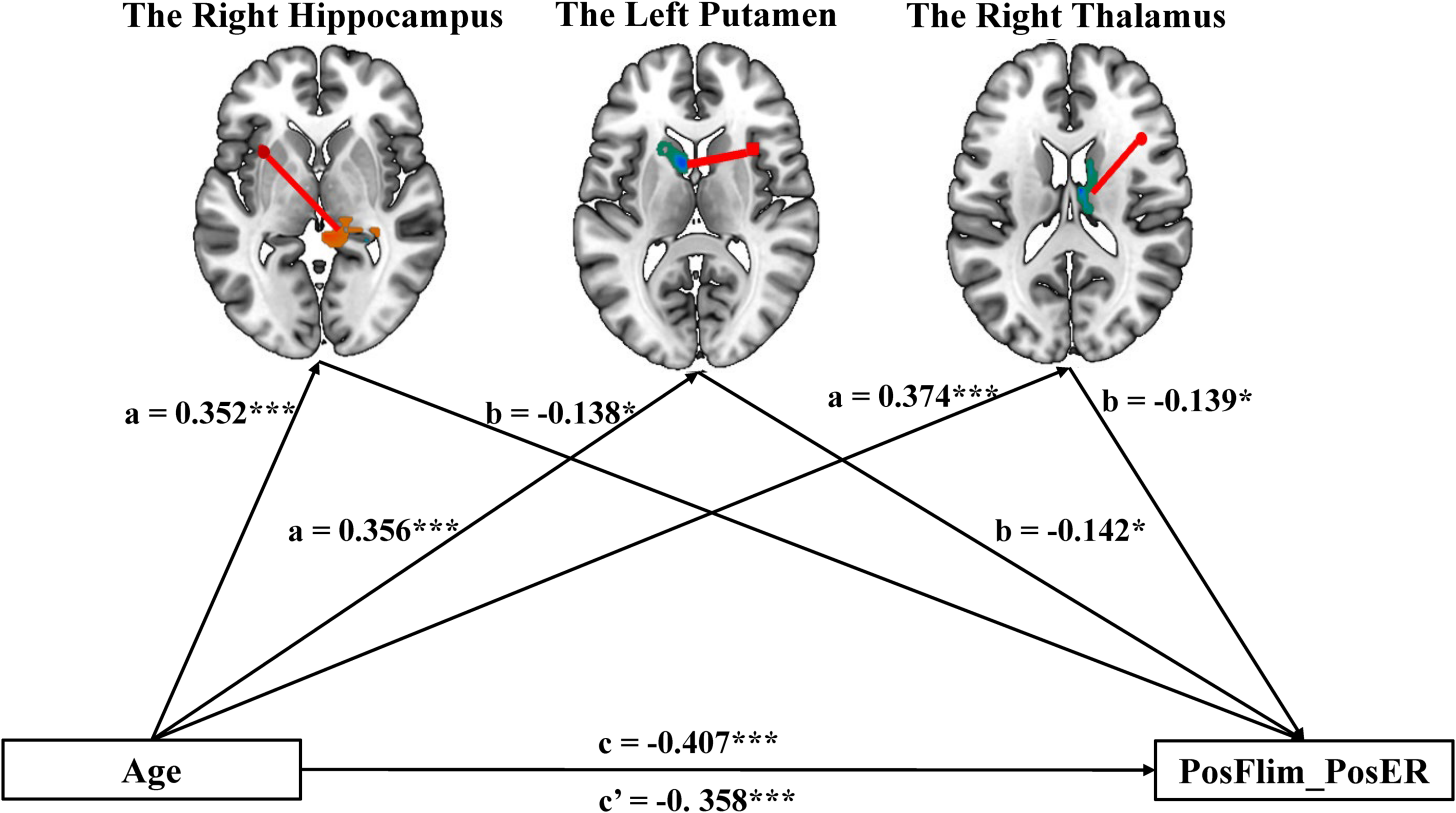
Results of mediation analysis. The mediating effect of age on positive emotional reactivity to watching positive films through anterior insula (AI). PosFlim_PosER, positive emotional reactivity to watching positive films. (*p < .05, **p < .01, ***p < .001).

Additionally, the results of the bootstrapping method confirmed the significance of the indirect effect of age through: (1) the rsFC of the left AI with the right hippocampus (95% CI = [−0.0117, −0.0007]; indirect effect = −0.0052; Table S5); (2) the rsFC of the right AI with the left putamen (95% CI = [−0.0127, −0.0003]; indirect effect = −0.0054, Table S6); and (3) the rsFC of the right AI with the right thalamus (95% CI = [−0.0128, −0.0002]; indirect effect = −0.0056, Table S7). The indirect effect of the rsFC of the left AI with the right hippocampus, and rsFC of the right AI with the left putamen and the right thalamus, separately, accounted for 11.93%, 12.39%, and 12.82%, respectively, of the total variance in positive emotional reactivity to watching positive films. Figure 7 illustrates the mediation model, along with standardized path coefficients.

## 4 DISCUSSION

This study has investigated the relationship between age and emotional reactivity, as well as the mediating role of the rsFCs in each subregion of the insula. We first found that as age increases, both negative and positive emotional reactivity decreases significantly with age, especially positive emotional reactivity. Then, we found that the rsFC of the left AI with the right hippocampus, as well as the rsFCs of the right AI with the striatum and the thalamus mediated the relationship between positive emotional reactivity and age.

Our research emphasizes the crucial role of the AI in age‐related cognitive functions, particularly emotional reactivity. Previous research has suggested that IS serves as a physiological basis for emotional processes, has a significant positive correlation with emotional reactions (MacCormack et al., 2020), and diminishes with age (Davey et al., 2021; Dunn et al., 2010; Lautenbacher et al., 2017; Schandry, 1981). Our study builds upon this foundation by revealing a concurrent decrease in emotional reactivity with age. Additionally, the findings show that the insula not only plays a role as a hub of the interoceptive awareness system (Zhao et al., 2023), but also plays a pivotal role in the decline of emotional reactivity with age. This suggests the possibility that, as individuals age, they become less sensitive to physiological changes triggered by emotional stimuli, leading to a greater reliance on cognitive assessments mediated by AI during emotional reactivity.

However, the decline in emotional reactivity with age appears to partially undermine the SST (Carstensen et al., 1999). SST emphasizes positive effects; that is, older adults tend to give primacy to achieving emotional gratification and avoiding or mitigating exposure to negative situations, which may shed light on the age differences in negative emotional reactivity found in this study. With regard to positive emotional reactivity, SST suggests that, as individuals age and perceive time as limited, they prioritize emotionally satisfying and meaningful goals, leading to more positive emotional reactivity (Truxillo et al., 2015). Although we did not observe the increase in positive emotional reactivity with age, we did observe that, as individuals age, whether facing negative or positive stimuli, their emotional reaction is reduced to a level similar to a neutral stimulus. In other words, within the framework of SST, our study may unveil an alternative emotion regulation strategy, in which older adults may choose to minimize extreme mood swings as they age, thereby maintaining emotional stability and achieving emotional well‐being. Lowering emotion to approach a neutral mood may be the reason why we did not observe an emotion regulation effect. However, this mode of emotion adaptation may also reflect an emotion regulation strategy that prioritizes emotional satisfaction while maintaining emotional stability.

The mediating effect was only observed in AI, possibly because AI is more connected to the limbic system than MI and PI, and thus may be involved in emotional processes (You et al., 2022). Another possible explanation is that the generation of emotional feelings requires a neural remapping of different features of the body state in the CNS, resulting from cognitive “appraisal” where the anterior insular cortex plays a key integrative role (Craig, 2009; Damasio & Carvalho, 2013). In older adults, there is a shift in activity from PI to AI during emotional reactivity; that is, AI is more involved in a variety of cognitive, perceptual, and socioemotional processes (Clos et al., 2014), with older adults more involved in mentalization, autobiographical memory, and self‐regulation during emotional responses, whereas younger adults are more dependent on PI's extensive involvement in visceral signaling.

Many studies have highlighted the role of the hippocampus in emotion regulation and its interaction with other brain regions involved in emotional processing and memory (Fanselow & Dong, 2010; Li et al., 2022; Yang & Wang, 2017). The anatomical structure of the hippocampus enables the brain to connect memory with emotions. According to lesion experiments, spatial memory depends on the dorsal hippocampus (DH), whereas stress response and emotional behavior depend on the ventral hippocampus (VH) (Frey et al., 2020). In addition, most studies have specifically divided the hippocampus into the dentate gyrus; the cornus ammonis (CA; CA1, CA2, and CA3); the entorhinal cortex; and the subiculum (von Ziegler et al., 2018). Our study found that the rsFC of the left AI with the right CH, which includes the ventral CA3—containing *N*‐methyl‐d‐aspartate receptors which play a key role in the antidepressant effects of the drug (Mastrodonato et al., 2018)—was positively correlated with age and negatively correlated with positive emotional reactivity.

One possible explanation is that lowering emotional reactivity to maintain emotional stability is a favorable strategy for individual emotion regulation. We suggested that the rsFC between the right AI and striatum decreases with age, which may be the main contribution to the decrease in the intensity of emotional reactivity with age. A study of twins found that the caudate volume was genetically correlated with six emotional characteristics (Choi et al., 2022). The ventral striatum, including the nucleus accumbens, is implicated in affective and motivational processing (Delgado, 2007). The insula, along with the striatum, is implicated in the regulation of emotions and subjective emotional states (Chen et al., 2018; Wei et al., 2022). Our findings further illustrate that the AI, along with the striatum, is involved in the integration of sensory, interoceptive, and emotional information to create a conscious evaluation of affective reactivity (Nanda et al., 2020). Another important observation is the consistent relationship between the GMV of the insula with age and emotional reactivity. Consistent with previous studies, we found that the GMV of the insula has a negative correlation with age. We then found a positive relationship between the GMV in the insula and positive emotional reactivity. This result suggests that the GMV of the insula is not only related to age but also to positive emotional reactivity. Although no mediating effect was found, this result could still provide us with a basis for explaining the relationship between age, emotional reactivity, and functional connectivity in the insula. Further exploration would need to separate the insula into subregions for detailed analysis.

There have been relatively few studies on functional connectivity between the thalamus and the insula, and this study shows that the rsFC of the right AI with the thalamus plays a crucial role in emotional reactivity and emotional processing. The thalamus is also involved in the generation of subjective emotional reactivity, as evidenced by its connectivity with the prefrontal cortex and anterior cingulate (Saarimäki et al., 2023). The present study extends this view to the rsFC between the thalamus and the right AI. While this study has focused on a general population sample, in anxiety and depression populations a dysfunctional connection between the thalamus and insula has been observed, highlighting the importance of this connection in emotional dysregulation (Liu & Graybiel, 1998).

Several considerations and challenges should be noted for future research on this topic. First, although we suggested that individual emotional reactivity and its decrease with age are related to the decrease in IS, we did not simultaneously measure IS with age. Hence, future research could continue to expand and supplement the current study from this perspective. Second, our observations of mediation effects are more exploratory, and we have only obtained individual resting‐state scan data. Future studies could recruit subjects to complete task‐state scans to further explore whether the changes in functional connectivity of the insula still have a mediating effect on the process of individual emotional reactivity. Finally, limited by the current dataset, our study focused on individuals over 18 years old, and future research could explore the relationships presented by different age groups, especially children and adolescents, if such datasets are available.

## 5 CONCLUSION

In sum, the current study used a population‐derived sample from across the human lifespan to investigate the age effect and neural correlates of individual emotional reactivity under the ecologically valid film‐based paradigm. We found that individuals tend to lower their emotional reactivity to maintain emotional stability with age. Whereas the rsFC between the left AI and the right hippocampus increased with age, the rsFCs between the right AI and striatum and thalamus decreased with age, sustaining the age‐related decrease in positive emotional reactivity. Future research should aim to replicate these findings in diverse population samples and consider task‐state scans to further elucidate the mediating effects of insula connectivity on emotional reactivity.

## CONFLICT OF INTEREST STATEMENT

The authors declare no competing financial interests.

## Supporting information


**DATA S1** Supporting Information.

## Data Availability

The data that support the findings of this study are openly available in Cambridge Centre for Ageing and Neuroscience at https://www.cam-can.or.
