# Supplementary material for: Age‐related positive emotional reactivity decline associated with the anterior insula based resting‐state functional connectivity
Source: Hum Brain Mapp. 2024 Feb 9;45(2):e26621. doi: 10.1002/hbm.26621 (PMC10858337; doi:10.1002/hbm.26621)
Supplement: Supplementary file 1 — DATA S1 Supporting Information. [file HBM-45-e26621-s001.docx]

Supplementary

Age-related positive emotional reactivity decline associated with the anterior insula based resting state functional connectivity

Lijing Niu^1^, Xiaoqi Song^1,3,4^, Qian Li^1^, Lanxin Peng^1^, Haowei Dai^1^, Jiayuan Zhang^1^, Keyin Chen^1^, Tatia M.C. Lee^3,4,5^, Ruibin Zhang^1,2^

^1^ Laboratory of Cognitive Control and Brain Healthy, School of Public Health, Southern Medical University, Guangzhou, PRC China

^2^ Department of Psychiatry, Zhujiang Hospital, Southern Medical University, Guangzhou, PRC China

^3^ State Key Laboratory of Brain and Cognitive Sciences, The University of Hong Kong, Hong Kong, SAR China

4 Laboratory of Neuropsychology and Human Neuroscience, The University of Hong Kong, Hong Kong, SAR China

5 Center for Brain Science and Brain-Inspired Intelligence, Guangdong-Hong Kong-Macao Greater Bay Area, Guangzhou, China

# Correspondence and request for materials should be addressed to:

Ruibin Zhang, Department of Psychology, School of Public Health, Southern Medical University (e-mail: [ruibinzhang@foxmail.com](mailto:ruibinzhang@foxmail.com))

**Methods**

**Overview of CAM-CAN data collection**

The aim of the Cambridge Centre for Ageing and Neuroscience (Cam-CAN) project is to identify the neural mechanisms underpinning successful cognitive ageing ([Shafto et al., 2014](#_ENREF_2); [Taylor et al., 2017](#_ENREF_3)). The data collection of the project was conducted in three stages. The project recruited a population-based cohort of 3000 adults aged 18 and over into stage 1, where they completed an interview including health and lifestyle questions, a core cognitive assessment, and a self-completed questionnaire of lifetime experiences and physical activity. Of those interviewed, 700 participants aged 18-87 continue to stage 2 where they undergo cognitive testing and provide measures of brain structure and function (e.g., structural image MRI, resting state fMRI, task-based fMRI). In stage 3, a subset of 280 adults returns for targeted neuroimaging experiments across a range of cognitive domains in four MRI and two MEG sessions (e.g., nine fMRI tasks, and six MEG tasks). The participants of stage 3 were divided into four groups, each engaging in two MRI sessions and one MEG session.

It was noticed that the resting-state scan in stage 3 had a duration of 5 minutes (compared to 8 minutes and 40 seconds in stage 2) and occurred between two task-related scans. For example, the resting-state scan of the fMRI session 3 was conducted after the completion of the Stop-Signal Go/No-Go MRI scan, and before the Emotional Memory encoding MRI scan ([Shafto et al., 2014](#_ENREF_2)). The scanning results may be influenced by the tasks ([Gaviria et al., 2021](#_ENREF_1)). Therefore, we only access the resting-state data of stage 2 (details of the session are outlined in Table S1).

Table S1 Stage 2 session content

| Stage 2 | | |  |
| --- | --- | --- | --- |
| Session | Measure | Modality | Approx.duration(mins) |
| Session 1 |  |  |  |
|  | Weight | Physiological | 2 |
|  | Hight | Physiological | 2 |
|  | Blood Pressure | Physiological | 11 |
|  | Visual short-term memory | Behavioural | 35 |
|  | Emotion expression recognition | Behavioural | 20 |
|  | T1-weighted structural image | MRI | 5 |
|  | T2-weighted structural image | MRI | 5 |
|  | Diffusion-Weighted Images | MRI | 10 |
|  | Magnetisation Transfer Ratio images | MRI | 5 |
|  | Resting-state T2*-weighted | fMRI | 9 |
|  | Movie watching T2*-weighted | fMRI | 8 |
|  | Sensorimotor task T2*-weighted | fMRI | 9 |
|  | Field maps | fMRI | 1 |
| Session2 |  |  |  |
|  | Face recognition: familiar faces | Behavioural | 10 |
|  | Face recognition: unfamiliar faces | Behavioural | 10 |
|  | Fluid Intelligence | Behavioural | 20 |
|  | Hotel task | Behavioural | 20 |
|  | Sentence comprehension | Behavioural | 30 |
|  | Resting-state | MEG | 9 |
|  | Sensorimotor task | MEG | 12 |
| Session 3a |  |  |  |
|  | Emotional reactivity and regulation | Behavioural | 55 |
|  | Force matching | Behavioural | 14 |
|  | Motor learning | Behavioural | 21 |
|  | Picture-picture priming | Behavioural | 20 |
|  | Proverb comprehension | Behavioural | 5 |
|  | Tip-of-the-tongue | Behavioural | 10 |
| Session 3b |  |  |  |
|  | Emotional memory | Behavioural | 90 |
|  | Picture-picture priming | Behavioural | 20 |
|  | Proverb comprehension | Behavioural | 5 |
|  | Tip-of-the-tongue | Behavioural | 10 |

**Participants exclusion criteria**

The stage 1 interview, held at participants' homes, is primarily conducted as an interview with computerized scripting for question asking and response recording. Measures obtained during this interview are utilized to screen participants for further engagement. To proceed to stage 2, participants must meet the following criteria:

(1) Be cognitively healthy, with MMSE scores above 24.

(2) Not have MRI safety, comfort, or medical contraindications, such as having various kinds of non-MRI compatible medical implants (cardiac pacemaker, cochlear implants, etc.) or magnetic foreign objects (e.g. shrapnel) close to the head, being pregnant, being claustrophobic, or being unable to lie still for the necessary length of time (approximately one hour). Criteria for MRI and MEG participation are defined by Standard Operating Procedures set by the Medical Research Council Cognition and Brain Sciences Unit (MRC-CBSU).

(3) Not have MEG contraindications that could affect data collection, such as extensive dental work (e.g. permanent brace).

(4) Not have other conditions including serious head injury, current drug abuse, or current serious psychiatric condition (e.g. bipolar, schizophrenic).

(5) Not have poor hearing which could affect the ability to participate in experiments (failing to hear 35 dB in either ear).

(6) Not have poor English or English which is extremely subordinate to another language, which could affect participation in experiments (i.e. those whose native language is not English or who are not bilingual English-speakers from birth).

**The resting state data acquisition**

MR imaging data were acquired using a 3T Siemens TIM Trio scanner with a 32-channel head coil. To assess intrinsic (passive) aspects of neural connectivity, T2*-weighted fMRI data are acquired while participants rest with their eyes shut using a Gradient-Echo Echo-Planar Imaging (EPI) sequence. A total of 261 volumes are acquired, each containing 32 axial slices (acquired in descending order), slice thickness of 3.7mm with an interslice gap of 20% (for whole brain coverage including cerebellum; TR = 1970 milliseconds; TE = 30 milliseconds; flip angle = 78 degrees; FOV = 192mm × 192mm; voxel-size = 3mm × 3mm × 4.44mm) and acquisition time of 8 minutes and 40 seconds.

**Results**

**Table S2.** The mediating effect of the rsFC in the left AI and right hippocampus on the relationship between age and positive emotional reactivity.

|  | Age→PosFilm_PosResctivity | | | | |
| --- | --- | --- | --- | --- | --- |
|  | *B* | *SE* | *β* | *t* | *p* |
| Gender | 0.415 | 0.238 | 0.105 | 1.746 | 0.082 |
| Mean FD | 3.405 | 1.368 | 0.170 | 2.490 | 0.013 |
| Age | -0.044 | 0.007 | -0.407 | -5.937 | <0.001 |
|  | Age→Left AI-Right Hippocampus | | | | |
|  | *B* | *SE* | *β* | *t* | *p* |
| Gender | -0.052 | 0.021 | -0.145 | -2.471 | 0.014 |
| Mean FD | 0.129 | 0.121 | 0.071 | 1.065 | 0.288 |
| Age | 0.003 | 0.001 | 0.352 | 5.266 | <0.001 |
|  | Age→Left AI-Right Hippocampu→PosFilm_PosExperiences | | | | |
|  | *B* | *SE* | *β* | *t* | *p* |
| Gender | 0.336 | 0.239 | 0.085 | 1.404 | 0.162 |
| Mean FD | 3.602 | 1.361 | 0.180 | 2.646 | 0.009 |
| Age | -0.038 | 0.008 | -0.358 | -4.982 | <0.001 |
| Left AI-  Right Hippocampus | -1.521 | 0.724 | -0.138 | -2.102 | 0.037 |

Note: PosFilm_PosReactivity refers to the positive emotional reactivity of subjects watching positive films; Left AI-Right Hippocampus refers to the rsFC in the left AI and right hippocampus.

**Table S3.** The mediating effect of the rsFC in the right AI and left putamen on the relationship between age and positive emotional reactivity.

|  | Age→PosFilm_PosExperience | | | | |
| --- | --- | --- | --- | --- | --- |
|  | *B* | *SE* | *β* | *t* | *p* |
| Gender | 0.415 | 0.238 | 0.105 | 1.746 | 0.082 |
| Mean FD | 3.405 | 1.368 | 0.170 | 2.490 | 0.013 |
| Age | -0.044 | 0.007 | -0.407 | -5.937 | <0.001 |
|  | Age→Right AI-Left Putamen | | | | |
|  | *B* | *SE* | *β* | *t* | *p* |
| Gender | 0.008 | 0.020 | 0.025 | 0.406 | 0.685 |
| Mean FD | -0.011 | 0.117 | -0.007 | -0.095 | 0.924 |
| Age | -0.003 | 0.001 | -0.356 | -5.154 | <0.001 |
|  | Age→Right AI-Left Putamen→PosFilm_PosExperiences | | | | |
|  | *B* | *SE* | *β* | *t* | *p* |
| Gender | 0.401 | 0.236 | 0.101 | 1.701 | 0.090 |
| Mean FD | 3.424 | 1.356 | 0.171 | 2.524 | 0.012 |
| Age | -0.038 | 0.008 | -0.356 | -4.973 | <0.001 |
| Right AI-  Left Putamen | 1.669 | 0.749 | 0.142 | 2.228 | 0.027 |

Note: PosFilm_PosReactivity refers to the positive emotional reactivity of subjects watching positive films; Right AI-Left Putamen refers to the rsFC in the right AI and left putamen.

**Table S4.** The mediating effect of the rsFC in the right AI and right thalamus on the relationship between age and positive emotional reactivity.

|  | Age→PosFilm_PosExperiences | | | | |
| --- | --- | --- | --- | --- | --- |
|  | *B* | *SE* | *β* | *t* | *p* |
| Gender | 0.415 | 0.238 | 0.105 | 1.746 | 0.082 |
| Mean FD | 3.405 | 1.368 | 0.170 | 2.490 | 0.013 |
| Age | -0.044 | 0.007 | -0.407 | -5.937 | <0.001 |
|  | Age→Right AI-Right Thalamus | | | | |
|  | *B* | *SE* | *β* | *t* | *p* |
| Gender | 0.013 | 0.020 | 0.040 | 0.666 | 0.506 |
| Mean FD | 0.006 | 0.113 | 0.004 | 0.054 | 0.957 |
| Age | -0.003 | 0.001 | -0.374 | -5.446 | <0.001 |
|  | Age→Right AI-Right Thalamus→PosFilm_PosExperiences | | | | |
|  | *B* | *SE* | *β* | *t* | *p* |
| Gender | 0.393 | 0.236 | 0.099 | 1.664 | 0.097 |
| Mean FD | 3.395 | 1.357 | 0.170 | 2.501 | 0.013 |
| Age | -0.038 | .008 | -0.355 | -4.923 | <0.001 |
| Right AI-  Right Thalamus | 1.680 | 0.776 | 0.139 | 2.165 | 0.031 |

Note: PosFilm_PosReactivity refers to the positive emotional reactivity of subjects watching positive films; Right AI-Right Thalamus refers to the rsFC in the right AI and right thalamus.

**Table S5.** The mediating effect of the rsFC in the left AI and right hippocampus examination by bootstrap

|  | *Effect* | *SE* | *LL 95%CI* | *UL 95%CI* |
| --- | --- | --- | --- | --- |
| Indirect effect | -0.0052 | 0.0027 | -0.0117 | -0.0007 |
| Direct effect | -0.0384 | 0.0077 | -0.0536 | -0.0232 |

**Table S6.** The mediating effect of the rsFC in the right AI and right putamen examination by bootstrap

|  | *Effect* | *SE* | *LL 95%CI* | *UL 95%CI* |
| --- | --- | --- | --- | --- |
| Indirect effect | -0.0054 | 0.0032 | -0.0127 | -0.0003 |
| Direct effect | -0.0382 | 0.0077 | -0.0534 | -0.0231 |

**Table S7.** The mediating effect of the rsFC in the right AI and right thalamus examination by bootstrap

|  | *Effect* | *SE* | *LL 95%CI* | *UL 95%CI* |
| --- | --- | --- | --- | --- |
| Indirect effect | -0.0056 | 0.0032 | -0.0128 | -0.0002 |
| Direct effect | -0.0381 | 0.0077 | -0.0533 | -0.0228 |

**References**

Gaviria, J., Rey, G., Bolton, T., Delgado, J., Van De Ville, D., & Vuilleumier, P. (2021). Brain functional connectivity dynamics at rest in the aftermath of affective and cognitive challenges. *Human Brain Mapping*, *42*(4), 1054-1069. <https://doi.org/10.1002/hbm.25277>

Shafto, M. A., Tyler, L. K., Dixon, M., Taylor, J. R., Rowe, J. B., Cusack, R., Calder, A. J., Marslen-Wilson, W. D., Duncan, J., Dalgleish, T., Henson, R. N., Brayne, C., & Matthews, F. E. (2014). The Cambridge Centre for Ageing and Neuroscience (Cam-CAN) study protocol: a cross-sectional, lifespan, multidisciplinary examination of healthy cognitive ageing. *BMC Neurology*, *14*, 204. <https://doi.org/10.1186/s12883-014-0204-1>

Taylor, J. R., Williams, N., Cusack, R., Auer, T., Shafto, M. A., Dixon, M., Tyler, L. K., Cam, C., & Henson, R. N. (2017). The Cambridge Centre for Ageing and Neuroscience (Cam-CAN) data repository: Structural and functional MRI, MEG, and cognitive data from a cross-sectional adult lifespan sample. *NeuroImage*, *144*(Pt B), 262-269. <https://doi.org/10.1016/j.neuroimage.2015.09.018>
